# ChIP-seq and transcriptome analysis of the OmpR regulon of *Salmonella enterica* serovars Typhi and Typhimurium reveals accessory genes implicated in host colonization

**DOI:** 10.1111/mmi.12111

**Published:** 2012-12-19

**Authors:** Timothy T Perkins, Mark R Davies, Elizabeth J Klemm, Gary Rowley, Thomas Wileman, Keith James, Thomas Keane, Duncan Maskell, Jay C D Hinton, Gordon Dougan, Robert A Kingsley

**Affiliations:** 1 The Wellcome Trust Sanger Institute The Wellcome Trust Genome Campus, Hinxton CambridgeCB10 1SA UK; 2 School of Biological Sciences University of East Anglia NorwichNR4 7TJ UK; 3 Department of Veterinary Medicine University of Cambridge Madingley Road CambridgeCB3 0ES UK; 4 Institute of Integrative Biology University of Liverpool Crown Street LiverpoolL69 7ZB UK

## Abstract

OmpR is a multifunctional DNA binding regulator with orthologues in many enteric bacteria that exhibits classical regulator activity as well as nucleoid-associated protein-like characteristics. In the enteric pathogen *Salmonella enterica*, using chromatin immunoprecipitation of OmpR:FLAG and nucleotide sequencing, 43 putative OmpR binding sites were identified in *S. enterica* serovar Typhi, 22 of which were associated with OmpR-regulated genes. Mutation of a sequence motif (TGTWACAW) that was associated with the putative OmpR binding sites abrogated binding of OmpR:6×His to the tviA upstream region. A core set of 31 orthologous genes were found to exhibit OmpR-dependent expression in both *S.* Typhi and *S.* Typhimurium. *S.* Typhimurium-encoded orthologues of two divergently transcribed OmpR-regulated operons (SL1068–71 and SL1066–67) had a putative OmpR binding site in the inter-operon region in *S.* Typhi, and were characterized using *in vitro* and *in vivo* assays. These operons are widely distributed within *S. enterica* but absent from the closely related *Escherichia coli*. SL1066 and SL1067 were required for growth on *N*-acetylmuramic acid as a sole carbon source. SL1068–71 exhibited sequence similarity to sialic acid uptake systems and contributed to colonization of the ileum and caecum in the streptomycin-pretreated mouse model of colitis.

## Introduction

OmpR is a DNA binding protein that, with the cognate sensor EnvZ, co-ordinates transcriptional response to environmental factors including osmotic stress in many enteric bacteria (Forst and Roberts, 1994). OmpR/EnvZ are central to the adaptive response to the intestinal environment (Giraud *et al*., 2008), in part because of the distinct osmolyte composition of the lumen. As many as 125 genes in *Escherichia coli* (Oshima *et al*., 2002) and 305 genes in *Salmonella* Typhi (Perkins *et al*., 2009) have been implicated in OmpR/EnvZ-dependent expression. The OmpR regulon includes genes from the ‘ancestral’ core genome shared with many enteric bacteria as well as genes of the accessory genome. The latter include virulence-associated loci such as the *viaB* locus that encodes Vi polysaccharide biosynthesis genes, and genes encoded on *Salmonella* pathogenicity island 2 (SPI-2) via its regulation of *ssrAB* (Pickard *et al*., 1994; Feng *et al*., 2003; Perkins *et al*., 2009). OmpR-regulated orthologues in diverse enteric bacteria define the ancestral regulon and include porin genes such as *ompF* and *ompC*. However, the OmpR regulon exhibits considerable plasticity and can include genes of the ancillary genome acquired by horizontal gene transfer, many of which are involved in host–pathogen interactions. The acquisition of such genes and the ability to express them appropriately on moving from the intestinal lumen to the intracellular compartment were likely key features in the evolution of *Salmonella* (Bäumler, 1997; Groisman and Ochman, 1997).

The genus *Salmonella* consists of more than 2500 serotypes that exhibit diverse host range and pathogenicity (Bäumler *et al*., 1997; Popoff *et al*., 2004). Most of the > 2500 serovars of *Salmonella enterica* have a relatively broad host range and are typically associated with gastroenteritis in human (Santos *et al*., 2001). In contrast, *S. enterica* serovar Typhi (*S.* Typhi) is highly host-adapted to cause the systemic disease typhoid specifically in human. *S.* Typhi can invade the intestinal mucosa but colonization of the intestine is relatively transient and rapid systemic dissemination can follow leading to typhoid. This distinct pathogenesis is driven at least in part by horizontally acquired genes that are required for virulence, including the OmpR-regulated *viaB*, encoding the Vi polysaccharide antigen (Pickard *et al*., 1994). The OmpR regulon includes both *Salmonella* pathogenicity island 1 (SPI-1) and SPI-2, mediated through *ssrAB* expression. The integration of such horizontally acquired genes into existing regulons is a recurring theme in the evolution of pathogenesis. The mechanism by which OmpR regulates gene expression is not fully understood. It has been proposed that OmpR has only weak specificity for DNA binding (Head *et al*., 1998; Rhee *et al*., 2008) and that it may have both a classical site-specific impact on gene expression through recruitment of RNA polymerase and additional nucleoid-associated protein (NAP)-like properties that may also impact global gene expression (Cameron and Dorman, 2012).

In this study we combine RNA-seq and ChIP-seq together with *in vitro* and *in vivo* phenotyping to define the interaction of OmpR with the chromosome and characterize two novel OmpR-regulated operons that are part of the *S. enterica* ancillary genome.

## Results

### Identification of candidate *S.* Typhi genes regulated by OmpR using ChIP-seq

We recently characterized the OmpR regulon of *S.* Typhi BRD948 using DNA microarray and RNA-seq (Perkins *et al*., 2009) (Table S1) identifying 208 genes by microarray and 305 genes by RNA-seq, that exhibited OmpR-dependent transcription during mid-log phase culture in rich media. In order to further characterize the OmpR regulon we used ChIP-seq to identify candidate genome regions that are preferentially associated with the OmpR protein in the *S.* Typhi genome. To this end a *S.* Typhi BRD948 derivative TT53.8 was constructed in which the 3′ end of *ompR* harboured an in-frame fusion with sequence encoding three repeats of the FLAG epitope (3×FLAG tag). TT53.8 expressed the fusion protein (OmpR::3×FLAG) in place of the wild-type OmpR protein from the native chromosomal location at single copy. To assess if this fusion protein had comparable function to wild-type OmpR, we indirectly monitored the expression of the *ompR*-dependent *viaB* locus in TT53 (Pickard *et al*., 1994). Agglutination of *S.* Typhi TT53 with anti-Vi antiserum was indistinguishable to that of *S.* Typhi BRD948 in low-salt and high-salt culture media (data not shown).

**Figure 1:**
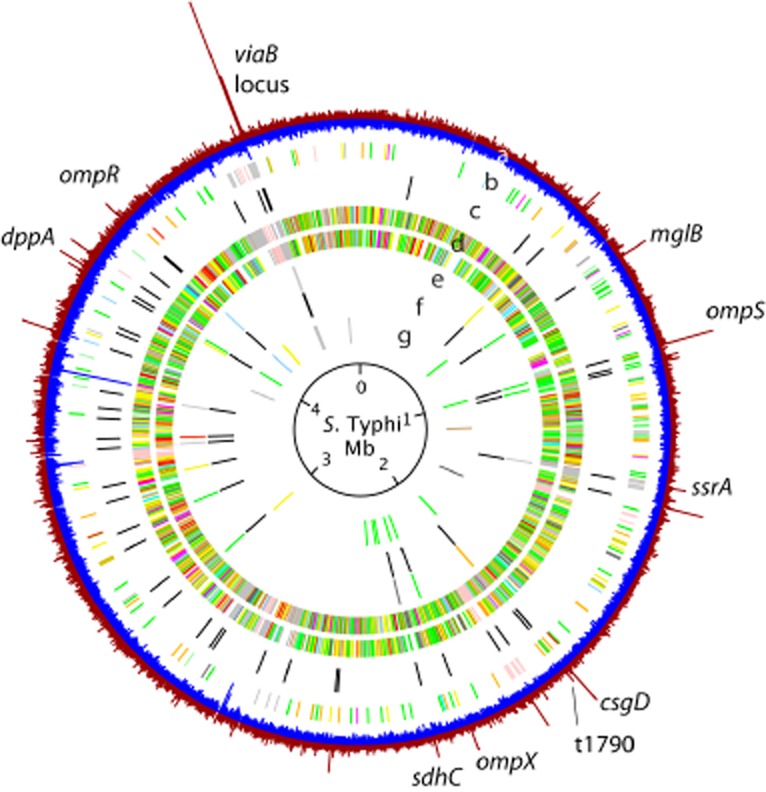
Circular plot of the *S.* Typhi Ty2 genome indicating ChIP-seq coverage and the position of OmpR-regulated genes. Concentric circular tracks indicate: (a) plot of the sequence coverage expressed as the number of standard deviations from the mean (*z*-score) with values > 0 (red) or < 0 (blue); (b) differentially regulated genes identified by microarray (Perkins *et al*., 2009); (c) significant ChIP-seq peaks defined as spanning ≥ 36 bp and with sequence read depth > 3 standard deviations (*z*-score) above the mean; (d) *S.* Typhi genes with colour coding indicating function: dark blue, pathogenicity/adaptation; black, energy metabolism; red, information transfer; dark green, membranes/surface structures; cyan, degradation of macromolecules; purple, degradation of small molecules; yellow, central/intermediary metabolism; light blue, regulators; pink, phage/IS elements; orange, conserved hypothetical; pale green, unknown function; brown, pseudogenes; (e) genes exhibiting OmpR-dependent expression that have a ChIP-seq peak in 5′ UTR region; (f) ChIP-seq peaks associated with differentially regulated genes; and (g) *S.* Typhi genes that exhibited OmpR-dependent expression in both microarray and RNA-seq experiments (Perkins *et al*., 2009).

*Salmonella* Typhi TT53.8 or BRD948 were grown to mid-log phase (OD_600_ = 0.6) and ChIP-seq was performed on DNA precipitated by anti-FLAG antibody. The normalized sequence depth at each base of the reference genome sequence was plotted as the number of standard deviations from the mean (*z*-score) to identify regions of significantly enriched sequence coverage (*z*-score > 3) and 43 ChIP-enriched peaks that were within intergenic regions were studied further (15 lay within annotated coding sequence and were excluded from further analysis) (Fig. 1, Table S2).

**Figure 2:**
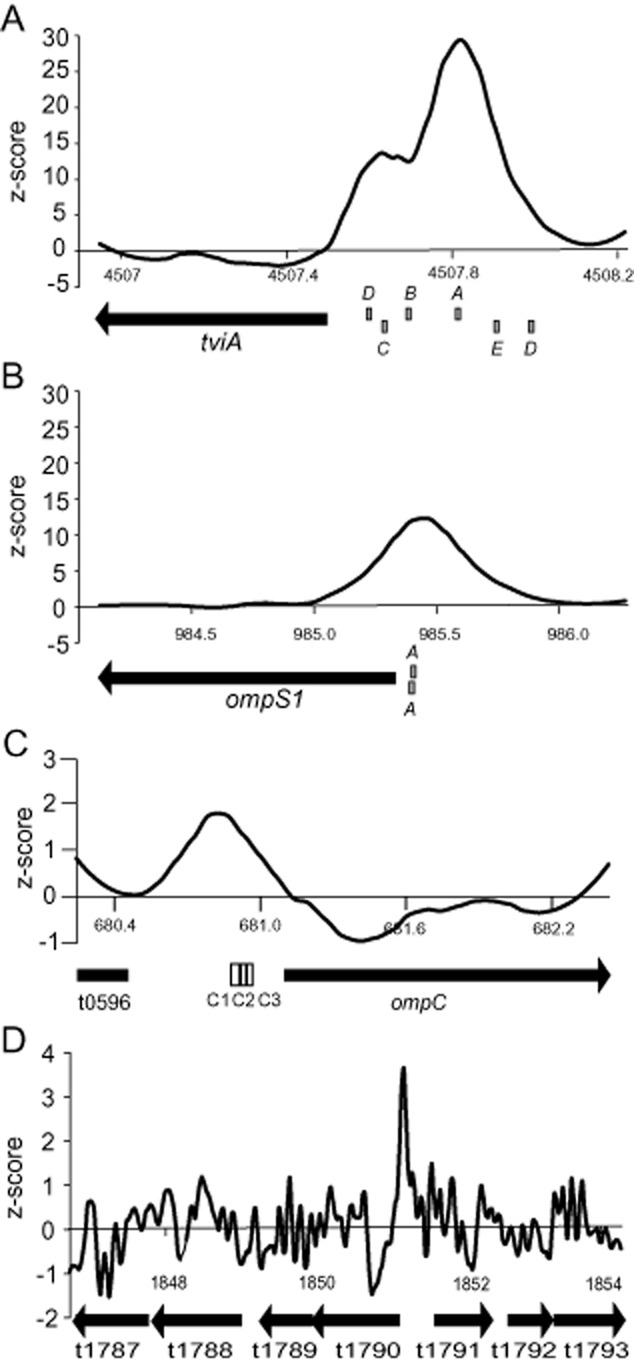
ChIP-seq coverage mapped to the *S.* Typhi Ty2 genome sequence in OmpR ChIP-seq. The *z*-score (number of standard deviations from the mean) is plotted at each base of the genome sequence in the 5′ UTR of (A) tviA, (B) ompS1, (C) between csgB and csgD and (D) t1787–1793. Motifs identified in this manuscript are indicated (grey boxes) labelled ‘A’ (TGTWACAW), ‘B’ (CTAGACTA), ‘C’ (AYGGCCTA), ‘D’ (AACTAACW) and ‘E’ (ATCTAGCS). OmpR binding sites in the ompC upstream region are indicated (open boxes) and labelled C1–3.

Twenty-two of the genes with a sequence enrichment peak in their upstream region also exhibited OmpR-dependent expression as determined by RNA-seq or microarray analysis (Perkins *et al*., 2009) (Fig. 1, Table 1). These included many previously identified OmpR-regulated genes such as *tviA* and *ompS1* (Fig. 2). Some genes associated with aerobic lifestyle such as citrate synthase (*gltA*) and succinate dehydrogenase C (*sdhC*) also contained enrichment peaks. A ChIP peak was identified in the intergenic region of the divergently transcribed operons encoding *stdA* and *dppA*. Another within the intragenic region of two divergently transcribed putative operons encoding genes t1787–1790 and t1791–93 (Table 2). Surprisingly, statistically significant peaks with a *z*-score > 3 were not identified in the well-characterized OmpR-regulated genes *ompF* and *ompC*, although a minor peak that fell just short of the statistical cut-off, mapped extensively to motifs (C1–3) implicated in OmpR binding (Fig. 2C). To determine if the C-terminal FLAG tag of OmpR impacted binding to the *ompC* or *ompF* promoter region we compared expression of these genes in the wild-type (BRD948) and *ompR*::FLAG strains (TT53.8) (Fig. S1). Expression of *tviB* and *ompF* was similar in these two strains but *ompC* was expressed at a significantly lower level in TT53.8. The degree to which *ompC* expression was decreased in TT53.8 compared to wild-type BRD948 was not as pronounced as that in a strain in which *ompR* was deleted (TT10) suggesting that some OmpR activity for the *ompC* promoter was retained in the epitope-tagged protein.

**Table 1:** *S.* Typhi genes that exhibit OmpR-dependent expression and have candidate OmpR binding sites in their 5′ UTR determined by ChIP-seq

Gene	Ty2 ID	CT18 ID	RNA-seq *ompR^+^*/*ompR^−^*	*P*-value	Microarray *ompR*^+^/*ompR*^−^	*P*-value	Ty2 annotation
*ackA*	t0527	STY2567	0.56	2.6 × 10^−2^	NS	NS	Acetate kinase
*mglB*	t0665	STY2424	2.0	9.4 × 10^−2^	2.17	3.3 × 10^−8^	d-galactose binding periplasmic protein
*ompS1*	t0883	STY2203	3.0 × 10^−1^	7.9 × 10^−3^	4.2 × 10^−1^	3.7 × 10^−4^	Outer membrane protein S1
*fliC*	t0918	STY2167	NS	NS	1.9	1.6 × 10^−3^	Flagellin
*ssrA*	t1260	STY1728	9.9 × 10^−2^	1.6 × 10^−3^	NS	NS	Putative two-component sensor kinase
–	t1790	STY1167	1.0 × 10^−1^	3.1 × 10^−2^	8.3	7.1 × 10^−8^	Hypothetical protein
–	t1791	STY1166	8.18	9.1 × 10^−2^	1.8	3.3 × 10^−5^	*N*-acetylmannosamine-6-phosphate 2-epimerase
*ompX*	t2055	STY0872	4.4 × 10^−1^	3.6 × 10^−3^	NS	NS	Outer membrane protein X
*sdhC*	t2144	STY0775	NS	NS	4.9	9.3 × 10^−10^	Succinate dehydrogenase
*gltA*	t2146	STY0773	NS	NS	6.8	6.4 × 10^−10^	Citrate synthase
*stdA*	t2940	STY3177	2.20 × 10^−1^	3.4 × 10^−2^	NS	NS	Probable fimbrial protein
–	t3197	STY3460	5.21 × 10^−1^	9.3 × 10^−2^	NS	NS	Tryptophan permease
*udp*	t3329	STY3591	3.8	7.5 × 10^−3^	NS	NS	Uridine phosphorylase
*purH*	t3455	STY3709	3.9 × 10^−1^	2 × 10^−2^	NS	NS	Bifunctional phosphoribosylaminoimidazolecarboxamide formyltransferase
*rplK*	t3478	STY3736	4.3 × 10^−1^	6.5 × 10^−3^	NS	NS	50S ribosomal protein L11
*typA*	t3613	STY3871	3.8 × 10^−1^	8.2 × 10^−2^	NS	NS	GTP binding protein
–	t3871	STY4154	2.2	9.4 × 10^−2^	NS	NS	Putative transcriptional regulator
*dppA*	t3885	STY4168	2.2	7.7 × 10^−3^	2.1	3.9 × 10^−7^	Periplasmic dipeptide transport protein
*ompR*	t4004	STY4294	1.1 × 10^−1^	6.3 × 10^−4^	3.6 × 10^−1^	1.9 × 10^−5^	Osmolarity response regulator
*tviA*	t4353	STY4662	3.8 × 10^−3^	1 × 10^−4^	3.8 × 10^−2^	4.7 × 10^−11^	Vi polysaccharide biosynthesis protein
–	t4354	STY4663	2.0 × 10^−1^	2.3 × 10^−2^	NS	NS	Hypothetical protein
–	t4357	STY4666	4.4 × 10^−1^	8.4 × 10^−3^	NS	NS	Probable phage integrase

RNA-seq data, normalized ratio (WT : Δ*ompR*) of sequence coverage determined by Illumina GA11 sequencing (Perkins *et al*., 2009).

Normalized ratio (WT : Δ*ompR*) of microarray (*n* = 3) fluorescent intensities from three biological replicates hybridized on four microarrays (Perkins *et al*., 2009).

NS, not significantly different.

**Table 2:** Novel OmpR-regulated genes in *S.* Typhi operons t1787–90 and t1791–93 and *S.* Typhimurium orthologues

*S.* Typhi Ty2 ID	*S.* Typhimurium orthologue (SL1344)	Log_2_ fold change *ompR*^+^/*ompR*^−^ *S.* Typhi	GC content (%)	Putative function
t1787	SL1071	4.03	46.8	Oxidoreductase
t1788	SL1070	2.86	45.1	Sialic acid transporter
t1789	SL1069	4.44	36.3	Secreted protein
t1790	SL1068	8.33	40.2	Sialic acid lyase
t1791	SL1067	1.78	51.4	*N*-acetylmannosamine-6-P epimerase
t1792	1.33	43.9
t1793	SL1066	1.21	47.0	SSS sialic acid transporter

Log_2_ fold change in transcript abundance determined by RNA-seq (Perkins *et al*., 2009).

### Identification of nucleotide sequence motifs associated with OmpR binding

To identify sequence motifs within the 43 intergenic ChIP-enriched sequence coverage peaks that may be involved in OmpR binding, nucleotide sequences were compared using the YMF algorithm (Sinha and Tompa, 2003), which identifies candidate binding sites by searching for statistically overrepresented motifs. Five eight-nucleotide motifs (*z*-score > 9.8, Table S2) were identified in 14 separate loci, with some loci containing multiple motifs. The motif TGTWACAW occurred 21 times, in 12 ChIP-enriched sequences including the *viaB* locus (5′ *tviA*) where it precisely coincided with the peak of sequence enrichment (Fig. 2). The maximum *z*-score of peaks associated with this motif was significantly greater (*P* = 0.0013, Student's *t*-test) than that of peaks without an identifiable motif (*P* = 0.0013, Student's *t*-test) (Fig. S2). The TGTWACAW motif was present in two copies in the upstream region of seven genes: *ompS1*, *csgD*, *sdhC*, *galP*, *dppA*, *pckA* and t4357. t4357 encodes a putative integrase encoded on a prophage ∼ 3.6 kbp upstream of *tviA*. A second motif AYGGCCTA was present in single copy in the upstream region of four loci: t0528, t1320, *dppA* and *tviA*. There was also a significant difference in the magnitude of ChIP sequence peaks containing motif AYGGCCTA compared with those with no identifiable motif (Student's *t*-test, *P* < 0.0001) (Fig. S2), suggesting a link between this sequence and the avidity of OmpR binding. None of the sequence motifs are present in the previously described C1–3 or F1–4 OmpR binding sites in *ompC* and *ompF* promoter regions respectively.

**Figure 3:**
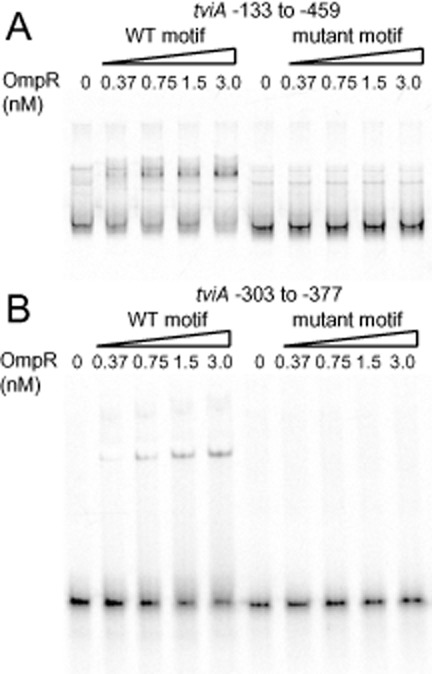
Electrophoretic mobility shift assay to determine binding of recombinant OmpR::6×His to the tviA upstream region. FAM-labelled double-stranded DNA fragments corresponding to the −133 to −459 (A) −303 to −377 (B) upstream region of tviA were mixed with increasing concentrations of phosphorylated recombinant OmpR::6×His protein and mobility monitored by electrophoresis on a 15% native polyacrylamide gel.

The largest enrichment peak (*z*-score = 16.23) was found upstream of *tviA* of the *viaB* locus, encoding four different candidate motifs: TGTWACAW, CTAGACTA, AYGGCCTA and AACTAACW (Table S2a). To find if the TGTWACAW motif was involved in binding of OmpR to the *tviA* upstream region, we used an electrophoresis mobility shift assay (EMSA) with phosphorylated recombinant OmpR::6×His protein and oligonucleotide probes. The probes comprised either *tviA* −133 to −460 or *tviA* −303 to −377 of the *tviA* upstream region. Arbitrary mutation of the TGTTACAA motif at the –341 to −348 position to GCTCGGAC resulted in abrogation of OmpR::6×His binding to either probe (Fig. 3). No binding of OmpR::6×His was observed with a probe containing the mutant motif sequence, suggesting that this motif is important for OmpR binding. Significantly, the TGTTACAA motif in the *tviA* upstream region also coincides with the genome sequence most highly overrepresented following ChIP enrichment (*z*-score = 30, Fig. 2).

### The OmpR regulons of *S.* Typhi and *S.* Typhimurium contain a core set of shared orthologous genes

**Figure 4:**
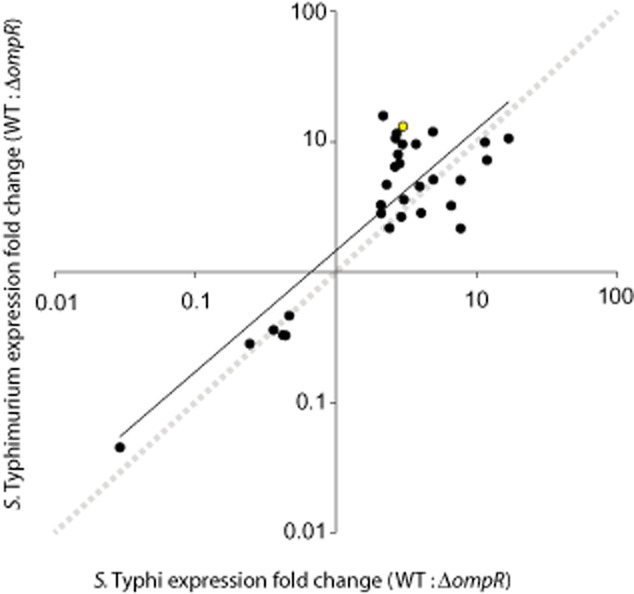
Comparison of OmpR-dependent expression of orthologous genes in *S.* Typhi and *S.* Typhimurium in microarray experiments. Fold change in expression of orthologous genes in wild-type (WT) compared to ΔompR strains of *S.* Typhi Ty2 and *S.* Typhimurium strain SL1344 are indicated (black circles). Orthologous genes t1789/SL1069 highlighted (yellow circle). The linear regression of the data points (solid black line, *R*^2^ = 0.73) and the line of equivalence (broken grey line) are indicated.

We next compared the previously unreported OmpR regulon of the broad host range *S.* Typhimurium strain SL1344 with that of the human restricted *S.* Typhi Ty2 (Perkins *et al*., 2009) to identify previously uncharacterized genes, controlled by OmpR in both pathogens. A total of 208 genes and 329 genes were expressed in an OmpR-dependent manner in *S.* Typhi and *S.* Typhimurium respectively. Of these, 31 orthologous genes were expressed in an OmpR-dependent manner in both serotypes (Table S3). OmpR-dependent expression levels of genes that were OmpR-regulated in both *S.* Typhi and *S.* Typhimurium showed a high degree of correlation (Fig. 4; *R*^2^ = 0.73) indicating strongly conserved regulation between the two serovars in this cohort of genes. OmpR-dependent genes found in both serotypes included *ompS1*, *ompC*, *sprB* and *ompR*, all of which showed decreased expression in the absence of OmpR. A number of genes were upregulated in the absence of OmpR, including the succinate dehydrogenase genes *sdhCDA* (Cunningham and Guest, 1998), fatty acid dehydrogenase genes *fadABI* (Campbell *et al*., 2003), *narK* (Rowe *et al*., 1994), required for nitrite extrusion, and the nitrite reductase gene *nrfA* (Clarke *et al*., 2008).

Potentially important differences in the expression of SPI-1, SPI-2 and flagellin secretion apparatus in *S.* Typhi and *S.* Typhimurium were also revealed by the transcriptomic data in *S.* Typhimurium; 28 SPI-1-associated genes exhibited decreased expression in the absence of OmpR, including the *sprB* gene. Furthermore, several genes associated with the flagella type III secretion system (*fliGHJLMOPR*) and 10 apparatus genes encoded on SPI-2 (*ssaA*, *ssaB*, *ssaGHIJKLT* and STM1410) also exhibited up to fivefold greater expression in the absence of a functional OmpR (Table S4). In contrast, the only known SPI-1 gene that was OmpR-dependent in *S.* Typhi was *sprB*, and this encodes a transcriptional regulator (Golubeva *et al*., 2012). However, it is important to note that the culture conditions employed in our studies are known to result in low expression of SPI-1 and SPI-2 genes and therefore the biological impact of the observed differences in OmpR regulation in these conditions is not clear.

### t1787–t1790 and t1791–1793 encode putative nutrient-scavenging systems

**Figure 5:**
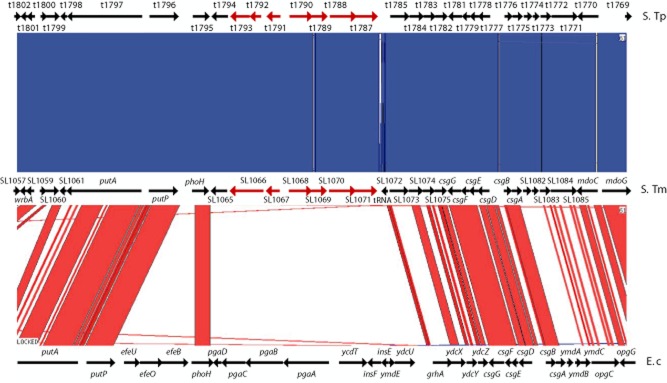
Genome alignment of the syntenic regions of *S.* Typhi Ty2, *S.* Typhimurium SL1344 and *E. coli* K12 containing novel OmpR-dependent operons t1787–t1790 and t1791–1793. Artemis comparison tool (ACT) view of the *S.* Typhi (S. Tp), *S.* Typhimurium (S. Tm) and *Escherichia coli* (E. c) in the t1787–t1790 and t1791–1793 genomic region. Red bars indicate regions of high sequence identity between like strands; blue bars indicate high sequence identity on opposite strands due to inversion.

We next characterized the function of t1787–t1790 and t1791–1793 using a number of *in vitro* and *in vivo* assays. In *S.* Typhimurium SL1344, genes SL1071–SL1068 (STM1133–STM1130) and SL1067–SL1066 (STM1129–STM1128) are orthologues of the *S.* Typhi t1787–t1790 and t1791–1793 genes respectively (Fig. 5). However, t1792 and t1793 of *S.* Typhi are present as a single open reading frame (SL1066) in *S.* Typhimurium, suggesting that these genes may represent fragments of a pseudogene in *S.* Typhi.

We considered that these operons may be involved in host–pathogen interactions since they were absent from the closely related species *E. coli* (strain K12) (Blattner *et al*., 1997) but were highly conserved within *S. enterica* serotypes exhibiting > 99% identity at the amino acid level in these pathogens (Fig. 5). The transcriptomic data showed that expression of at least two of the *S.* Typhimurium orthologues was OmpR-dependent, namely SL1068 (4.75-fold increase, *P* < 0.05, orthologue of t1791) and SL1069 (9.59-fold increase, *P* < 0.05, orthologue of t1789, Fig. 4). Proteins encoded by several of the genes in these operons exhibit similarity to proteins that have previously been implicated in sialic acid uptake and metabolism. An orthologue of SL1066 encoded by *S.* Typhimurium LT2 (STM1128) is a sodium solute symporter (SSS) family transporter of sialic acid and shares 44–48% identity at the amino acid level with similar transport systems in *Lactobacillus* spp. and *Staphylococcus* spp. (Severi *et al*., 2010). The STM1128 gene complemented a mutant *E. coli* lacking the sialic acid transporter NanT for growth on sialic acid as the sole source of carbon (Severi *et al*., 2010). SL1067 is a homologue of *nanE* (SL3309) that is encoded elsewhere on the SL1344 chromosome, sharing 69% identity at the amino acid level. SL1068 to SL1071 have previously been proposed to be orthologues of genes encoded by *E. coli* K12 (*nanM*, *nanC*, *yjhB* and *yjhC* respectively), some of which have been implicated in sialic acid metabolism (Severi *et al*., 2008). However, the genomic context for these genes is quite different in *E. coli* compared to *S.* Typhimurium and amino acid sequence identity ranges from just 22% for NanC to 62% for YhjC, considerably less than observed for orthologous proteins of *E. coli* and *S.* Typhimurium of approximately 90% (Parkhill *et al*., 2001). We therefore tested the hypothesis that genes within these operons were involved in utilization of acetylated amino sugars including sialic acid as a sole carbon source, and colonization of the murine host.

To this end, we determined the ability of *S.* Typhimurium SL1344 and the isogenic mutant derivatives RAK103 (ΔSL1067/SL1066) and RAK105 (ΔSL1068–SL1071) to grow on M9 minimal media supplemented with one of several alternative acetylated amino sugars as the sole source of carbon. RAK103 (ΔSL1067/SL1066) was deficient in growth on *N-*acetylmuramic acid, a component of peptidoglycan, but grew normally on all other sole carbon sources tested (Table 3). A strain (SW738) in which SL1067/SL1066 were reintroduced onto the chromosome of strain RAK103 (ΔSL1067/SL1066) by phage-mediated transduction grew normally on *N-*acetylmuramic acid as a sole carbon source. No defect in growth on any of the carbon sources tested was observed for RAK105 including *N*-acetylneuraminic acid, a common sialic acid, possibly because this strain encodes a second sialic transport protein, NanT.

**Table 3:** Growth of *S.* Typhimurium RAK113 (wild-type) or strains RAK103 and RAK105 on M9 minimal media agar supplemented with alternative sole carbon sources

Carbon source	Wild type	ΔSL1067–SL1066	ΔSL1068–SL1071
No carbon source	**−**	**−**	**−**
Glucose	**++**	**++**	**++**
Pectin	**−**	**−**	**−**
Galacturonic acid	**−**	**−**	**−**
Muramic acid	**−**	**−**	**−**
Cytidine sialic acid	**+**	**+**	**+**
*N*-acetylmuramic acid	**++**	**−**	**++**
*N*-acetylneuraminic acid	**++**	**++**	**++**

The surface of the agar was inoculated with each strain cultured on LB agar and incubated for 48 h at 37°C. Growth was assessed and recorded as no growth (−), growth retarded relative to that with glucose as a sole carbon source (+) and comparable growth to that with glucose as a sole carbon source (++).

Experimental infections of mice with *S.* Typhimurium are a common surrogate model for typhoid fever (Tsolis *et al*., 1999). To determine if these OmpR-regulated genes contribute to the pathogenesis of *S.* Typhimurium in mice we carried out competitive infection experiments between the fully virulent *S.* Typhimurium RAK113 and mutant derivatives harbouring deletions within each operon (RAK105 ΔSL1068–SL1071, orthologues of t1787–t1790 and RAK103 ΔSL1067/SL1066, orthologues of t1791–3) (Table 4). Initially, groups of C57BL/6 mice were inoculated orally with 1 × 10^8^ cfu of an equal mixture of RAK103 or RAK105 and RAK113. No significant difference in the ratio of each derivative was observed in the caecum, ileum, Peyers patch, spleen or liver (Table 4).

**Table 4:** Competitive infection of C57BL/6 mice

	Caecum			Ileum					Mesenteric lymph nodes							Liver									Spleen										
Log_10_ ratio	SE	Log_10_ ratio	SE	Log_10_ ratio	SE	Log_10_ ratio	SE	Log_10_ ratio	SE
Typhoid infection model										
WT : ΔSL1068–71	−0.26	1.40	0.55	1.25	0.65	0.80	−0.02	0.33	0.07	0.41
WT : ΔSL1066–67	−0.50	0.62	0.08	0.56	−0.41	0.36	−0.25	0.15	−0.21	0.18
Colitis infection model										
WT : ΔSL1066–67 (RAK103)	−0.18	0.10	−0.14	0.16	−	−	−	−	−	−
WT : ΔSL1068–71 (RAK105)	**−0.39**	0.12	−0.36***	0.09	−	−	−	−	−	−
RAK105 complemented	−0.08	0.20	−0.23	0.26	−	−	−	−	−	−

For the typhoid infection model, groups of five C57BL/6 mice were inoculated with 1 × 10^8^ cfu containing an equal mixture of either RAK103 (ΔSL1067/SL1066, orthologues of t1791–3) or RAK105 (ΔSL1068–SL1071, orthologues of t1787–t1790) and RAK113 (*phoN*::*cat*). For the colitis infection model, groups of five C57BL/6 mice pretreated with 1 mg of streptomycin sulphate 24 h prior to inoculation with 1 × 10^3^ cfu containing an equal mixture of either RAK103 (ΔSL1067/SL1066, orthologues of t1791–3) or RAK105 (ΔSL1068–SL1071, orthologues of t1787–t1790) and RAK113 (*phoN*::*cat*).

* *P* < 0.05,

** *P* < 0.005.

SE, standard error.

The data were calculated from the combined data from two independent experiments each of which individually had comparable outcomes.

Since *S.* Typhimurium normally causes inflammatory diarrhoea manifesting as gastroenteritis we determined if these genes are required for colonization and growth during a robust inflammatory response in the intestinal tract. To this end we performed mixed inoculum experiments in the streptomycin-pretreated mouse model of colitis (Hapfelmeier *et al*., 2004; Hapfelmeier and Hardt, 2005). Groups of streptomycin-pretreated mice were inoculated with 1 × 10^3^ cfu of an equal mixture of *S.* Typhimurium RAK103 or RAK105 and RAK113. Four days post inoculation RAK103 was present in similar proportion to RAK113 (Table 4). However, RAK113 was present in approximately threefold greater numbers in the caecum of mice compared with RAK105 (Table 4). This decrease in fitness specifically in the inflamed gut was statistically significant, and reproducible. Furthermore, when the SL1067/SL1066 genes were reintroduced into RAK105 by phage-mediated transduction giving rise to strain SW771, the ability to compete successfully with the wild-type RAK113 in colonization of the caecum in streptomycin-pretreated mice was restored (Table 4).

## Discussion

Transcriptional regulons have been defined using DNA microarrays and more recently by RNA-seq approaches. Observed changes in transcript abundance can be directly or indirectly related to a regulator protein binding either within an operator or at a secondary regulatory site. We have combined measurement of transcript abundance with a direct assay of OmpR binding using ChIP-seq to gain a more complete understanding of the regulon and identify novel genes within this network. Using this approach genome sequences that were enriched included many previously described OmpR-regulated genes (Fig. 1). The most highly enriched sequences were upstream of the *viaB* locus (*tviA*) (Fig. 2A). Furthermore, there was considerable enrichment in the 5′ UTR of *ompS1* (Fig. 2B), *ompR* and between the divergently transcribed *csg* operons. These observations provided proof of principle that the ChIP-seq approach identified well-known OmpR-regulated genes. Perhaps surprisingly, substantial enrichment peaks were not observed in the 5′ UTR of the *ompC* and *ompF* genes, even though these are known to be regulated by OmpR (Rhee *et al*., 2008). A minor peak that did map to previously identified OmpR binding sites (C1–3) was present, but fell below the criteria used for peak identification. The reason for the lack of enrichment peaks associated with the *ompC* and *ompF* genes is not known, but may be related to the specific culture conditions used in this study resulting in incomplete phosphorylation of OmpR or due to interference from the C-terminal FLAG epitope tag. The presence of a C-terminal FLAG epitope had little impact on expression of the *tviB* and *ompF* genes but the *ompC* gene exhibited decreased expression, suggesting that the epitope may impact binding sites differently. Therefore, it is possible that all OmpR binding sites were not identified in this study.

Specific binding of OmpR is thought to depend at least in part on short nucleotide sequence motifs in the 5′ UTR region of genes within the regulon (Huang *et al*., 1994; Harlocker *et al*., 1995; Rhee *et al*., 2008), although the dependence on specific sequence is markedly less pronounced than for another two-component regulator of *Salmonella*, PhoP (Harari *et al*., 2010). Specific recognition of these motifs by OmpR is dependent on the phosphorylation state of the regulator and subsequent positive regulation of transcription results from direct interaction with RNA polymerase. A number of motifs have been proposed based on sequence similarity in the 5′ UTR of the *ompC* and *ompF* genes of *E. coli*, and DNAase footprint analysis. However, the lack of specificity for OmpR binding to these motifs is shown by the absence from the upstream sequence of other OmpR-regulated genes. We used the YMF algorithm to identify sequences that were statistically overrepresented within enriched sequences following immunoprecipitation. While no such motifs were identified in 28 of 43 enrichment peaks using this approach, the motif (TGTWACAW) was present in 12 enrichment peaks and appeared in multiple copies (two or three copies) in seven of these regions. The motif TGTTACAA was present precisely at the point of greatest ChIP enrichment in the *tviA* upstream region determined by sequencing. Furthermore, this sequence was critical for binding of recombinant OmpR–6×His *in vitro* using an EMSA approach. A total of four additional motifs were also identified and generally where these were present they were in the 5′ UTR of genes that also contained the common motif TGTWACAW. This suggested there may be a functional link between these sequences.

A total of 31 orthologous pairs of genes showed OmpR-dependent expression in both *S.* Typhi and *S.* Typhimurium. Many more genes were OmpR-dependent in either *S.* Typhi or *S.* Typhimurium. The reason for this distinction is not clear but may be related to differences in the phosphorylation state of EnvZ, the OmpR cognate sensor kinase, that has been reported between these two serotypes (Oropeza and Calva, 2009). OmpR has pleiotropic effects on the homeostasis of the bacterial cell and these may manifest differently in Typhi and Typhimurium due to the overall differences in genotype.

Facultative anaerobic bacteria such as *E. coli* and *Salmonella* are thought to occupy a niche in the mucus layer close to the intestinal epithelium. Here they scavenge monosaccharides produced from the hydrolysis of complex polysaccharides and dietary fibre by anaerobic bacterial members of the microbiota (Chang *et al*., 2004). However, pathogenic bacteria such as *Salmonella* (Stecher *et al*., 2007) can induce a strong inflammatory response that results in a decrease in the population of many components of the microbiota that not only alters the available nutrients (Stecher *et al*., 2008) but also available respiratory electron acceptors (Winter *et al*., 2010). Two divergently transcribed operons that were differentially expressed on inactivation of the *ompR* gene and contained a candidate OmpR binding site were predicted to be involved in scavenging and transport of alternative carbon sources. The predicted product of t1787–t1790 (SL1071–SL1068) had sequence similarity to sialic acid transport and metabolism systems. However, genetic deletion of SL1071–SL1068 did not impact on the utilization of *N*-acetylneuraminic acid (sialic acid) as a sole carbon source under the conditions tested, probably due to the presence of other sialic acid transport system, such as NanA/NanT in *S.* Typhimurium (Plumbridge and Vimr, 1999). The proteins encoded by t1791–3 (SL1067–1066) are also predicted to be involved in sialic acid metabolism. SL1067 exhibited homology with NanE, an *N*-acetylmannosamine-6-phosphate epimerase, and SL1066 orthologue has been reported to complement a *nanT* mutant of *E. coli* for growth in sialic acid as a sole source of carbon (Severi *et al*., 2010). However, deletion of these genes did not detectably impact utilization of sialic acid *in vitro*, presumably because of the presence of *nanT* and *nanE*. However, deletion of these genes resulted in the inability to use a related acetylated carbon compound, *N-*acetylmuramic acid, as a sole source of carbon during culture *in vitro*. Furthermore, although SL1068–SL1071 were not obviously required for colonization of the murine host in conventional mixed inoculum experiments, in the colitis model there was a reproducible and statistically significant decrease in the ability of RAK105 ΔSL1068–SL1071 to colonize the caecum of streptomycin-pretreated mice in competition with the wild-type parent. *S.* Typhimurium RAK103 was indistinguishable from the RAK105 SL1067–SL1066 locus in the ability to colonize the murine host.

Sialic acid has several potential impacts on host–pathogen interactions. It can be utilized as a carbon or nitrogen source, and is used by *Haemophilus influenzae* to modify LPS in order to evade detection by the host immune system (Severi *et al*., 2005; 2007), although this has not been reported in enteric pathogens to date. Nutrient content of the intestine is impacted by the microbial community because of the complex interplay in catabolism of complex nutrients in the luminal contents (Bertin *et al*., 2012). However, it is likely that nutrient availability is altered as a result of the inflammatory response induced by *Salmonella* during infection, concomitant with disturbance of the normal microbiota (Stecher *et al*., 2007). Indeed, it was recently reported that *Salmonella* can use host-derived ethanolamine as a carbon source and respiratory electron acceptor following the switch to anaerobic respiration in the inflamed intestine (Thiennimitr *et al*., 2011). Our findings suggest that additional OmpR-regulated genes may contribute to nutrient scavenging in the inflamed intestine.

## Experimental procedures

### Bacterial culture and strains

*Salmonella* Typhi was cultured routinely in LB broth with aromatic amino acids and pABA supplements as described previously (Lowe *et al*., 1999). Growth media were supplemented with antibiotics as appropriate at final a concentration of 0.05 mg l^−1^ kanamycin or 0.03 mg l^−1^ chloramphenicol. A strain in which the *ompR* gene is replaced by the *aph* gene encoding kanamycin resistance has been described previously (Kingsley *et al*., 2003). To construct a chromosomally encoding *ompR*::3×FLAG, overlap extension PCR was employed to create a sequence encoding an in-frame 3×FLAG peptide at the C-terminus of the *ompR* gene. This was complicated by the overlapping start–stop codon of the *ompB* locus (Parkhill *et al*., 2001). The Shine–Dalgarno sequence of the *envZ* gene, predicted to be encoded in the *ompR* ORF, was encoded downstream of the stop codon after the 3×FLAG sequence. This sequence was cloned into the suicide vector pWT12 and the strain TT53 made by allelic exchange (Turner *et al*., 2006). Primers used were as follows: CGTCAGGCAAACGAACTGCC, 5′ to 3′ *ompR* bases (364.383), CCGTCATGGTCTTTGTAGTCTGCTTTAGAACCGTCCGGTA (full reverse primer sequence 5′ to 3′), GACTACAAAGACCATGACGGTGATTATAAAGATCATGATATCGATTACAAGGATGACGATGACAAGTAGGTACCGGACGGTTCTAAAGC [concatenated primers are 5′ to 3′ forward (1:69 FLAG + 1:20)], CGAAACGCAGGCGGCACG [reverse for *envZ* is 5′ to 3′ (213:230)]. A strain designated RAK105 in which the SL1068–SL1071 genes of SL1344 were replaced by the *aph* gene was constructed using oligonucleotides 5′ accataagatcactaatgatgaagctttactccaattgtatttcttcgcTGTGTAGGCTGGAGCTGCTTC 3′ and 5′ cataagcgcagcgccaccggccaataacaccaccatccggctttcaattCATATGAATATCCTCCTTAG 3′ to amplify with the pKD4 plasmid template. A strain designated RAK103 in which the SL1067–SL1066 genes of SL1344 were replaced by the *aph* gene was constructed using oligonucleotides 5′ cgcgttggcgtcaccgtatgctgtgtcggtatagcgtggtatcatgaaaTGTGTAGGCTGGAGCTGCTTCG 3′ and 5′ agacataacataaaacggagcaaaacttcaaatatataaggcggaactggCATATGAATATCCTCCTTAG 3′ to amplify with the pKD4 plasmid template. In all cases the mutation was retransduced into *S.* Typhimurium SL1344 using bacteriophage P22 in order to decrease the chances of the accumulation unlinked mutations during the passaging of bacteria during mutation construction. Strains (SW738 and SW771) in which the wild-type copy of genes SL1066 and SL1067 or SL1068–1071 was replaced in strains RAK103 (ΔSL1066–1067::*aph*) and RAK105 (ΔSL1068–1071::*aph*), respectively, were constructed using phage-mediated transduction. In order to select for transductants in this region a *cat* gene was introduced in the intergenic region of SL1071 and SL1072 using oligonucleotide primers 5′ cgcaaagtaaaactcactgaaattcttggctaaaattgaaagccgGTGTAGGCTGGAGCTGCTTCG 3′ and 5′ ccggtctacataagcgcagcgccaccggccaataacaccaccatcCATATGAATATCCTCCTTAG 3′. The *cat* gene was then introduced into *S.* Typhimurium strain RAK105 by P22 transduction and chloramphenicol-resistant transductants selected on LB + Cm culture medium. Transductants that were resistant to chloramphenicol but sensitive to kanamycin were identified by replica plating on culture media containing the appropriate antibiotics. One such transductant was designated SW771 and the replacement of the *aph* gene with the wild-type SL1068–1071 confirmed by PCR amplification.

### Expression analyses using microarray data and RNA-seq

Bacterial strains were cultured to OD_600_ = 0.6 and immediately fixed with RNAprotect (Qiagen) and harvested. The pellet was dried and RNA isolated using SV RNA isolation kit (Promega) according to manufacturer's instructions; however, elutions were performed using DEPC-treated water (Ambion). Dye incorporation, microarray design and analysis were performed as described previously (Kelly *et al*., 2004). RNA-seq data were described previously (Perkins *et al*., 2009). For *S.* Typhimurium microarrays, strain SL1344 and variants were cultured shaking at 250 r.p.m. in a New Brunswick Innova 3100 water bath at 37°C in 25 ml of fresh LB medium inoculated with a 1:100 dilution from an overnight bacterial culture. Three biological replicates were performed for each strain, and RNA was extracted at an optical density at 600 nm of 0.6 (mid-exponential phase). RNA was extracted using Promega's SV 96 total RNA purification kit. RNA quality was assessed on an Agilent 2100 Bioanalyser. Transcriptomic analyses were performed on a SALSA microarray that contained the 5000 open reading frames (ORFs) identified from the sequence of *S. enterica* serovar Typhimurium SL1344, as described previously (Balbontin *et al*., 2006). Hybridization, microarray scanning and data analysis were all performed as described previously (Kelly *et al*., 2004), using a false-discovery rate of 0.05. The expression data have been deposited in the NCBI GeneExpression Omnibus http://www.ncbi.nlm.nih.gov/geo/query/acc.cgi?token=pbkdfwskomsowpq&acc=GSE35938 and are accessible through GEO Series Accession Number GSE35938. All microarray data are MIAME-compliant.

### ChIP-seq

*Salmonella* Typhi *ompR*::3×FLAG (strain TT53) and *S.* Typhi BRD948 were cultured in LB broth to OD_600_ = 0.6, lysed, incubated with 1% formaldehyde at 37°C for 20 min to cross-link DNA with protein then quenched with glycine (ph7) to a final concentration of 0.5 M. Cells were harvested and washed twice in TBS and lysed by osmotic shock. Genomic DNA was then sheered by sonication to an average size of 300 bp and immunoprecipitated using anti-3×FLAG monoclonal antibody (Sigma, F3165) as previously described (Pfeiffer *et al*., 2007) using the Protein G Immunoprecipitation kit (Sigma). Eluates were then treated with pronase (0.8 mg ml^−1^, Sigma) at 65°C overnight. The nucleotide sequence of genomic DNA fragments was determined by Illumina GAII paired-end sequencing with read length 36 bp and mapped to the *S.* Typhi Ty2 whole genome sequence (AE014613). Sequence data were mapped to the *S.* Typhi Ty2 genome using the same parameters as previously described (Perkins *et al*., 2009), without assigning the sequence reads to each strand. Plots were *z*-score-normalized, in order to indicate the number of standard deviations above or below the mean for each datum point, and the differences between the untagged *S.* Typhi Ty2 and *ompR*::3×FLAG-tagged associated DNA sequences determined. Plots were then read into the genome browser tool Artemis (Rutherford *et al*., 2000). The Peakfinder function was used to determine enrichment for *OmpR*::3×FLAG bound DNA sequences. The Peakfinder function (36 bp window and *z*-score cut-off score set to 3) identified 58 peaks. Due to the background noise of the mapped sequence data plots and low stringency of the Peakfinder conditions, identified peaks were then filtered manually. Sites of DNA enrichment present within a predicted or known CDS were ignored unless there were multiple similar sites nearby, reducing the total number of analysed peaks to 43. Enriched sequences were then input to the motif finding algorithm YMF with the length of motif set to eight nucleotides and with a maximum of two redundant bases (Sinha and Tompa, 2003).

### RNA extraction, reverse transcription-PCR (RT-PCR) and real-time PCR

RNA was extracted from *S.* Typhi using a Fast RNA Blue Kit (MP Biomedicals) according to the instructions of the manufacturer. RNA samples (40 μg) were DNase I (Thermo Scientific) treated in a 100 μl volume and diluted to 100 ng μl^−1^. RNA samples were reverse transcribed and used as the template for Real-Time PCR with Express One-Step SYBR GreenER (Invitrogen) in a 20 μl total reaction volume. Real-Time PCR was performed using a StepOnePlus Real-Time PCR System (Applied Biosystems) with the oligonucleotides (Sigma) ATATGTTGGGCTTCCTCTGG and TTCAGATAACGAGCCTCACG (*tviB*), TTGATGGCCTGCACTACTTC and TGGTTGCCCTGAATCTGATA (*ompC*), GAAACGCAGATTAACACCGA and ACTTCCGCGTATTTCAAACC (*ompF*) and TACCTGCTGGCGGAGATTA and ATACCATGCTGATGCAGAGAA (*waaY*). Data were analysed by using the comparative C_T_ method where target gene transcription of each sample was normalized to the C_T_ of the *waaY* transcript.

### Electrophoretic mobility shift assay (EMSA)

For preparation of recombinant OmpR–6×His *S.* Typhi genomic DNA was PCR-amplified using oligonucleotide primers 5′ CATGCCATGGaagagaattataagattctgg 3′ and 5′ CCGCTCGAGtgctttagaaccgtccggtac 3′. The amplified DNA was cloned into pET28 vector into the NcoI and XhoI restriction sites giving rise to pTW1. One litre of *E. coli* BL21 pTW1 was cultured in Luria–Bertani containing 1 mM IPTG broth at 25°C to OD_600_ of 0.6. Cells were disrupted using a constant cell disruptor (Constant Systems), centrifuged at 23500 rcf and OmpR–6×His purified from the supernatant by affinity chromatography using nickel-resin chromatography. OmpR was phosphorylated with lithium potassium acetyl phosphate as previously described (Kenney *et al*., 1995). Double-stranded DNA probes were either PCR-amplified from *S.* Typhi genomic DNA using primers 5′ 6-FAM – AACGGGATTTTTACACAACAGAG 3′ and 5′ 6-FAM – AGTCATTATCCATATCTTTAATTTG 3′ (probe 1), or by annealing the oligonucleotides 5′ 6-FAM – TCAAAATAAGAATATTCCTAATCGTATTTGAAATAATCTGTTACAAATTTAATTGTTTGCACCTTTGGGGTTAAA 3′ and 5′ 6-FAM – TTTAACCCCAAAGGTGCAAACAATTAAATTTGTAACAGATTATTTCAAATACGATTAGGAATATTCTTATTTTGA 3′. Probes with mutated putative binding motif were generated by overlap extension PCR using the oligonucleotide primers 5′ catagaaaaggtacaagcaatatc 3′, 5′ caattaaat**gctcggac**gattatttcaaatacgattaggaatattc 3′, 5′ agtatcacccactacccagg 3′ and 5′ gaaataatc**gtccgagc**atttaattgtttgcacctttggg 3′, and subsequent amplification with 6-FAM labelled primers above. EMSA binding assay was performed in 10 mM Tris.HCl pH 7.2, 50 mM KCl, 5 mM MgCl_2_, 1 mM EDTA, 1 mM DTT, 1 mg ml^−1^ BSA, 0.001 mg of poly(dIdC) and 5% glycerol. 10 nM OmpR–6×His was incubated with various concentrations of 6-carboxyfluorescein (6-FAM)-labelled probe DNA shaking for 35 min at 30°C. Samples were separated on a 10% TBE polyacrylamide gel (Biorad) and 6-FAM-labelled nucleic acid imaged using a Typhoon 9200 (Amersham).

### Animal experiments

In all mouse experiments female, 7–8 week-old C57BL/6 mice (Charles River) were inoculated orally by gavage with *S.* Typhimurium suspended in PBS pH 7.4. For mixed inoculum experiments in order to distinguish the wild-type strain from the mutant test strains, a *cat* (chloramphenicol acetyltransferase, chloramphenicol resistance gene) was inserted in the *S.* Typhimurium SL1344 chromosome in a position that has previously been described to have no effect on colonization of the murine host (Kingsley *et al*., 2003; Winter *et al*., 2010) (*phoN* locus, strain RAK113). Groups of five mice were inoculated orally with a 1:1 (log_10_ = 0) of approximately 1 × 10^8^ cfu of strain RAK113 and the test strain. When mice were moribund (less than 80% body weight compared with day of inoculation) or on day 5 post inoculation, mice were culled and cfu of each strain in homogenized mesenteric lymph nodes (MLN), caecum, ileum, spleen and liver was determined by serial dilution in PBS pH 7.4 and plating on LB agar containing cloramphenicol and LB agar containing kanamycin. Serial 10-fold dilutions were plated on LB + Cm or LB + Km agar, as appropriate, to determine cfu per organ. The ratio of wild-type (strain RAK113) to test strain was transformed to log_10_ and to determine if these values were significantly different from the log_10_ of the input ratio (input ratio log_10_ = 0) was determined using the two-tailed Student's *t*-test in the Prism 4 software version 4.0c (Graph Pad). Value for *P* < 0.05 was considered significantly different.

### Minimal media growth assays

*Salmonella* Typhimurium SL1344 and isogenic mutant strains were grown overnight and washed three times in PBS and then plated onto M9 minimal media supplemented with l-histidine (for SL1344 growth) and 1% agar. The glucose carbon source was substituted with amino sugars. Wild-type controls were grown concomitantly on separate plates made from the same agar mix.

### Ethics statement

All animal procedures were performed in accordance with the United Kingdom Home Office Inspectorate under the Animals (Scientific Procedures) Act 1986. The Wellcome Trust Sanger Institute Ethical Review Committee granted ethical approval for these procedures.

## References

[b1] Balbontin R, Rowley G, Pucciarelli MG, Lopez-Garrido J, Wormstone Y, Lucchini S (2006). DNA adenine methylation regulates virulence gene expression in *Salmonella enterica* serovar Typhimurium. J Bacteriol.

[b2] Bäumler AJ (1997). The record of horizontal gene transfer in *Salmonella*. Trends Microbiol.

[b3] Bäumler AJ, Gilde AJ, Tsolis RM, Velden AWM, Ahmer BMM, Heffron F (1997). Contribution of horizontal gene transfer and deletion events to the development of distinctive patterns of fimbrial operons during evolution of *Salmonella* serotypes. J Bacteriol.

[b4] Bertin Y, Chaucheyras-Durand F, Robbe-Masselot C, Durand A, Foye A, Harel J (2012). Carbohydrate utilization by enterohaemorrhagic *Escherichia coli* O157:H7 in bovine intestinal content. Environ Microbiol.

[b5] Blattner F, Plunkett G, Bloch C, Perna N, Burland V, Riley M (1997). The complete genome sequence of *Escherichia coli* K-12. Science.

[b6] Cameron AD, Dorman CJ (2012). A fundamental regulatory mechanism operating through OmpR and DNA topology controls expression of *Salmonella* pathogenicity islands SPI-1 and SPI-2. PLoS Genet.

[b7] Campbell JW, Morgan-Kiss RM, Cronan JE (2003). A new *Escherichia coli* metabolic competency: growth on fatty acids by a novel anaerobic beta-oxidation pathway. Mol Microbiol.

[b8] Chang DE, Smalley DJ, Tucker DL, Leatham M, Norris WE, Stevenson SJ (2004). Carbon nutrition of *Escherichia coli* in the mouse intestine. Proc Natl Acad Sci USA.

[b9] Clarke TA, Mills PC, Poock SR, Butt JN, Cheesman MR, Cole JA (2008). *Escherichia coli* cytochrome c nitrite reductase NrfA. Methods Enzymol.

[b10] Cunningham L, Guest JR (1998). Transcription and transcript processing in the *sdhCDAB* – *sucABCD* operon of *Escherichia coli*. Microbiology.

[b11] Feng X, Oropeza R, Kenney LJ (2003). Dual regulation by phospho-OmpR of *ssrA**B* gene expression in *Salmonella* pathogenicity island 2. Mol Microbiol.

[b12] Forst SA, Roberts DL (1994). Signal transduction by the EnvZ–OmpR phosphotransfer system in bacteria. Res Microbiol.

[b13] Giraud A, Arous S, Paepe MD, Gaboriau-Routhiau V, Bambou JC, Rakotobe S (2008). Dissecting the genetic components of adaptation of *Escherichia coli* to the mouse gut. PLoS Genet.

[b14] Golubeva YA, Sadik AY, Ellermeier JR, Slauch JM (2012). Integrating global regulatory input into the *Salmonella* pathogenicity island 1 type III secretion system. Genetics.

[b15] Groisman EA, Ochman H (1997). How *Salmonella* became a pathogen. Trends Microbiol.

[b17] Hapfelmeier S, Hardt WD (2005). A mouse model for *S* Typhimurium-induced enterocolitis. Trends Microbiol.

[b16] Hapfelmeier S, Ehrbar K, Stecher B, Barthel M, Kremer M, Hardt WD (2004). Role of the *Salmonella* pathogenicity island 1 effector proteins SipA, SopB, SopE, and SopE2 in *Salmonella enterica* subspecies 1 serovar Typhimurium colitis in streptomycin-pretreated mice. Infect Immun.

[b18] Harari O, Park SY, Huang H, Groisman EA, Zwir I (2010). Defining the plasticity of transcription factor binding sites by deconstructing DNA consensus sequences: the PhoP-binding sites among gamma/enterobacteria. PLoS Comput Biol.

[b19] Harlocker SL, Bergstrom L, Inouye M (1995). Tandem binding of six OmpR proteins to the *ompF* upstream regulatory sequence of *Escherichia coli*. J Biol Chem.

[b20] Head CG, Tardy A, Kenney LJ (1998). Relative binding affinities of OmpR and OmpR-phosphate at the *ompF* and *ompC* regulatory sites. J Mol Biol.

[b21] Huang KJ, Schieberl JL, Igo MM (1994). A distant upstream site involved in the negative regulation of the *Escherichia coli ompF* gene. J Bacteriol.

[b22] Kelly A, Goldberg MD, Carroll RK, Danino V, Hinton JC, Dorman CJ (2004). A global role for Fis in the transcriptional control of metabolism and type III secretion in *Salmonella enterica* serovar Typhimurium. Microbiology.

[b23] Kenney LJ, Bauer MD, Silhavy TJ (1995). Phosphorylation-dependent conformational changes in OmpR, an osmoregulatory DNA-binding protein of *Escherichia coli*. Proc Natl Acad Sci USA.

[b24] Kingsley RA, Humphries AD, Weening EH, Zoete MRD, Winter S, Papaconstantinopoulou A (2003). Molecular and phenotypic analysis of the CS54 island of *Salmonella enterica* serotype Typhimurium: identification of intestinal colonization and persistence determinants. Infect Immun.

[b25] Lowe DC, Savidge TC, Pickard D, Eckmann L, Kagnoff MF, Dougan G, Chatfield SN (1999). Characterization of candidate live oral *Salmonella typhi* vaccine strains harboring defined mutations in *aroA**aroC*, and *htrA*. Infect Immun.

[b26] Oropeza R, Calva E (2009). The cysteine 354 and 277 residues of *Salmonella enterica* serovar Typhi EnvZ are determinants of autophosphorylation and OmpR phosphorylation. FEMS Microbiol Lett.

[b27] Oshima T, Aiba H, Masuda Y, Kanaya S, Sugiura M, Wanner BL (2002). Transcriptome analysis of all two-component regulatory system mutants of *Escherichia coli* K-12. Mol Microbiol.

[b28] Parkhill J, Dougan G, James KD, Thomson NR, Pickard D, Wain J (2001). Complete genome sequence of a multiple drug resistant *Salmonella enterica* serovar Typhi CT18. Nature.

[b29] Perkins TT, Kingsley RA, Fookes MC, Gardner PP, James KD, Yu L (2009). A strand-specific RNA-Seq analysis of the transcriptome of the typhoid bacillus *Salmonella typhi*. PLoS Genet.

[b30] Pfeiffer V, Sittka A, Tomer R, Tedin K, Brinkmann V, Vogel J (2007). A small non-coding RNA of the invasion gene island (SPI-1) represses outer membrane protein synthesis from the *Salmonella* core genome. Mol Microbiol.

[b31] Pickard D, Li J, Roberts M, Maskell D, Hone D, Levine M (1994). Characterization of defined *ompR* mutants of *Salmonella typhi**ompR* is involved in the regulation of Vi polysaccharide expression. Infect Immun.

[b32] Plumbridge J, Vimr E (1999). Convergent pathways for utilization of the amino sugars *N*-acetylglucosamine, *N*-acetylmannosamine, and *N*-acetylneuraminic acid by *Escherichia coli*. J Bacteriol.

[b33] Popoff MY, Bockemühl J, Gheesling LL (2004). Supplement 2002 (No. 46) to the Kauffmann-White scheme. Res Microbiol.

[b34] Rhee JE, Sheng W, Morgan LK, Nolet R, Liao X, Kenney LJ (2008). Amino acids important for DNA recognition by the response regulator OmpR. J Biol Chem.

[b35] Rowe JJ, Ubbink-Kok T, Molenaar D, Konings WN, Driessen AJ (1994). NarK is a nitrite-extrusion system involved in anaerobic nitrate respiration by *Escherichia coli*. Mol Microbiol.

[b1001] Rutherford K, Parkhill J, Crook J, Horsnell T, Rice P, Rajandream MA, Barrell B (2000). Artemis: sequence visualization and annotation. Bioinformatics.

[b36] Santos RL, Zhang S, Tsolis RM, Kingsley RA, Adams LG, Baumler AJ (2001). Animal models of *Salmonella* infections: enteritis versus typhoid fever. Microbes Infect.

[b40] Severi E, Randle G, Kivlin P, Whitfield K, Young R, Moxon R (2005). Sialic acid transport in *Haemophilus influenzae* is essential for lipopolysaccharide sialylation and serum resistance and is dependent on a novel tripartite ATP-independent periplasmic transporter. Mol Microbiol.

[b37] Severi E, Hood DW, Thomas GH (2007). Sialic acid utilization by bacterial pathogens. Microbiology.

[b39] Severi E, Muller A, Potts JR, Leech A, Williamson D, Wilson KS, Thomas GH (2008). Sialic acid mutarotation is catalyzed by the *Escherichia coli* beta-propeller protein YjhT. J Biol Chem.

[b38] Severi E, Hosie AH, Hawkhead JA, Thomas GH (2010). Characterization of a novel sialic acid transporter of the sodium solute symporter (SSS) family and *in vivo* comparison with known bacterial sialic acid transporters. FEMS Microbiol Lett.

[b41] Sinha S, Tompa M (2003). YMF: a program for discovery of novel transcription factor binding sites by statistical overrepresentation. Nucleic Acids Res.

[b43] Stecher B, Robbiani R, Walker AW, Westendorf AM, Barthel M, Kremer M (2007). *Salmonella enterica* serovar Typhimurium exploits inflammation to compete with the intestinal microbiota. PLoS Biol.

[b42] Stecher B, Barthel M, Schlumberger MC, Haberli L, Rabsch W, Kremer M, Hardt WD (2008). Motility allows *S* Typhimurium to benefit from the mucosal defence. Cell Microbiol.

[b44] Thiennimitr P, Winter SE, Winter MG, Xavier MN, Tolstikov V, Huseby DL (2011). Intestinal inflammation allows *Salmonella* to use ethanolamine to compete with the microbiota. Proc Natl Acad Sci USA.

[b45] Tsolis RM, Kingsley RA, Townsend SM, Ficht TA, Adams LG, Baumler AJ (1999). Of mice, calves, and men. Comparison of the mouse typhoid model with other *Salmonella* infections. Adv Exp Med Biol.

[b46] Turner AK, Nair S, Wain J (2006). The acquisition of full fluoroquinolone resistance in *Salmonella* Typhi by accumulation of point mutations in the topoisomerase targets. J Antimicrob Chemother.

[b47] Winter SE, Thiennimitr P, Winter MG, Butler BP, Huseby DL, Crawford RW (2010). Gut inflammation provides a respiratory electron acceptor for *Salmonella*. Nature.

