# Supplementary material for: ChIP-seq and transcriptome analysis of the OmpR regulon of Salmonella enterica serovars Typhi and Typhimurium reveals accessory genes implicated in host colonization
Source: Mol Microbiol. 2012 Dec 19;87(3):526–38. doi: 10.1111/mmi.12111 (PMC3586657; doi:10.1111/mmi.12111)
Supplement: Supplementary file 1 [file mmi0087-0526-SD1.pdf]

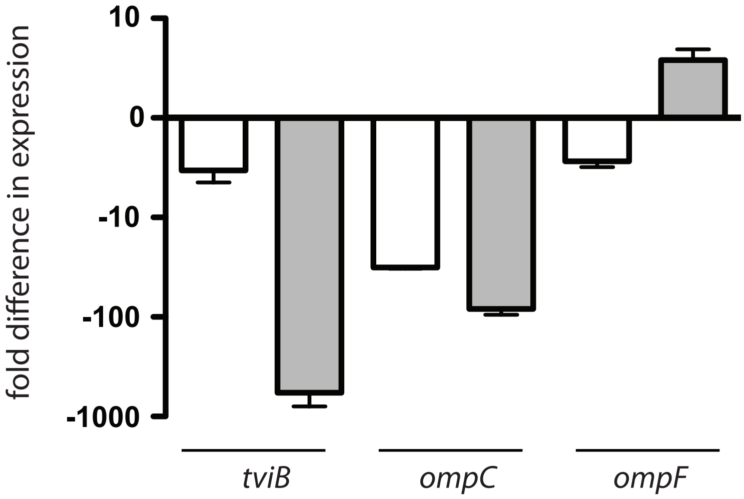

Figure S1. **Quantitative real-time PCR quantification of *tviB*, *ompC* and *ompF* transcript.** Relative transcript abundance in *S*. Typhi strain BRD948 (wild type *ompR*) relative to either strain TT53.8 (*ompR*::FLAG) (white bars) or strain BRD948 relative to TT10 (*ompR* mutant) (grey bars). Bars represent the mean of three 3 biological replicates with the standard deviation indicated.

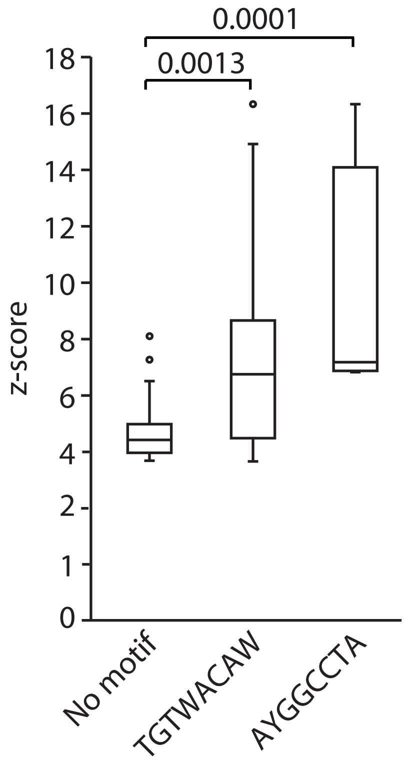

Figure S2. **Mean sequence coverage of ChIP-seq peaks containing candidate OmpR-binding motifs are greater than those with no identifiable motif.** The box plot indicates that the mean peak height (number of standard deviations from the mean sequence coverage, z-score) for peaks

containing either no identifiable sequence motif, or peaks associated with either motif TGTWACAW or AYGGCCTA. Values above box plots indicate p values calculated using Mann Whitney test.

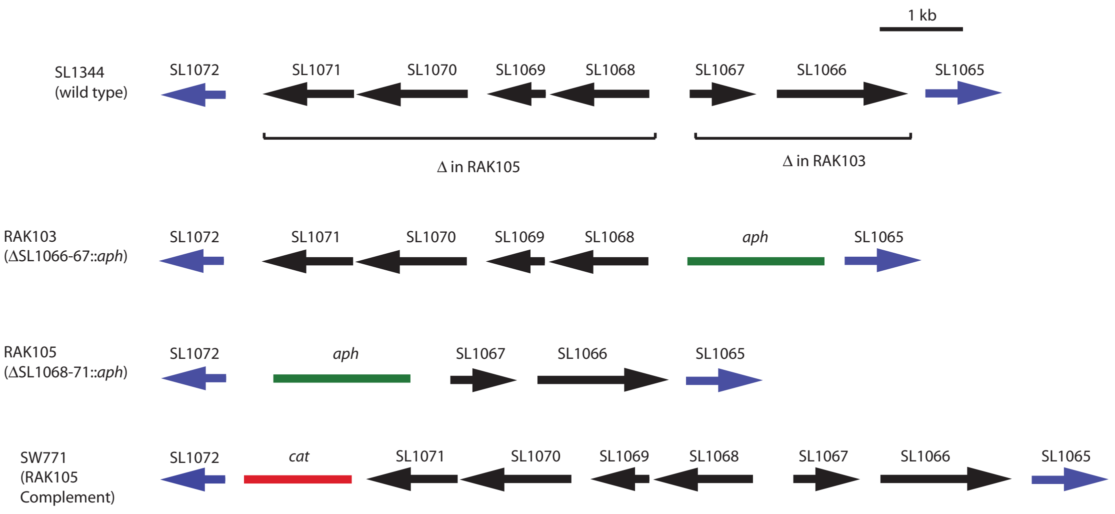

Figure S3. Summary of genotype of mutant and complement strains used in this study. *S*. Typhimurium genes are indicated (arrows) and solid bars indicate *cat* (red) or *aph* (green) genes that were used in allelic replacement experiments.

Supplementary file 1

peaks_42total_filtered_36bp_gt3.gff

A general feature format (.gff) file indicating the position of peaks of enriched sequence following ChIP in gff format. This file can be viewed in the Artemis or IGB genome browser software.

unknown_id EMBL/GenBank/SwissProt misc_binding 547334 547371
3.9227326 + . note "Auto-generated from User algorithm from min_zscored_normalised_chIP_combined.plot.txt plot; window size=3; score cut-off=3; from the peaks"
unknown_id EMBL/GenBank/SwissProt misc_binding 602000 602094
7.31772 + . note "Auto-generated from User algorithm from min_zscored_normalised_chIP_combined.plot.txt plot; window size=3; score cut-off=3; from the peaks"
unknown_id EMBL/GenBank/SwissProt misc_binding 750239 750390
6.4539347 + . note "Auto-generated from User algorithm from min_zscored_normalised_chIP_combined.plot.txt plot; window size=3; score cut-off=3; from the peaks"
unknown_id EMBL/GenBank/SwissProt misc_binding 985276 985761
8.895602 + . note "Auto-generated from User algorithm from min_zscored_normalised_chIP_combined.plot.txt plot; window size=3; score cut-off=3; from the peaks"
unknown_id EMBL/GenBank/SwissProt misc_binding 1013771 1013818
4.404567 + . note "Auto-generated from User algorithm from min_zscored_normalised_chIP_combined.plot.txt plot; window size=3; score cut-off=3; from the peaks"
unknown_id EMBL/GenBank/SwissProt misc_binding 1334195 1334368
4.953229 + . note "Auto-generated from User algorithm from min_zscored_normalised_chIP_combined.plot.txt plot; window size=3; score cut-off=3; from the peaks"
unknown_id EMBL/GenBank/SwissProt misc_binding 1382129 1382467
6.967168 + . note "Auto-generated from User algorithm from min_zscored_normalised_chIP_combined.plot.txt plot; window size=3; score cut-off=3; from the peaks"
unknown_id EMBL/GenBank/SwissProt misc_binding 1840063 1840542
7.7002826 + . note "Auto-generated from User algorithm from min_zscored_normalised_chIP_combined.plot.txt plot; window size=3; score cut-off=3; from the peaks"
unknown_id EMBL/GenBank/SwissProt misc_binding 1851257 1851295
4.4976363 + . note "Auto-generated from User algorithm

from min_zscored_normalised_chIP_combined.plot.txt plot; window size=3; score cut-off=3; from the peaks"
unknown_id EMBL/GenBank/SwissProt misc_binding 1956472 1956578 7.2103167 + . note "Auto-generated from User algorithm from min_zscored_normalised_chIP_combined.plot.txt plot; window size=3; score cut-off=3; from the peaks"
unknown_id EMBL/GenBank/SwissProt misc_binding 2117707 2117937 5.381847 + . note "Auto-generated from User algorithm from min_zscored_normalised_chIP_combined.plot.txt plot; window size=3; score cut-off=3; from the peaks"
unknown_id EMBL/GenBank/SwissProt misc_binding 2208487 2208530 4.350337 + . note "Auto-generated from User algorithm from min_zscored_normalised_chIP_combined.plot.txt plot; window size=3; score cut-off=3; from the peaks"
unknown_id EMBL/GenBank/SwissProt misc_binding 2208670 2208779 5.428038 + . note "Auto-generated from User algorithm from min_zscored_normalised_chIP_combined.plot.txt plot; window size=3; score cut-off=3; from the peaks"
unknown_id EMBL/GenBank/SwissProt misc_binding 2454505 2454541 3.762574 + . note "Auto-generated from User algorithm from min_zscored_normalised_chIP_combined.plot.txt plot; window size=3; score cut-off=3; from the peaks"
unknown_id EMBL/GenBank/SwissProt misc_binding 2461614 2461737 5.587058 + . note "Auto-generated from User algorithm from min_zscored_normalised_chIP_combined.plot.txt plot; window size=3; score cut-off=3; from the peaks"
unknown_id EMBL/GenBank/SwissProt misc_binding 2614253 2614289 3.6448805 + . note "Auto-generated from User algorithm from min_zscored_normalised_chIP_combined.plot.txt plot; window size=3; score cut-off=3; from the peaks"
unknown_id EMBL/GenBank/SwissProt misc_binding 2698630 2698667 4.4815617 + . note "Auto-generated from User algorithm from min_zscored_normalised_chIP_combined.plot.txt plot; window size=3; score cut-off=3; from the peaks"
unknown_id EMBL/GenBank/SwissProt misc_binding 2845064 2845108 4.427952 + . note "Auto-generated from User algorithm from min_zscored_normalised_chIP_combined.plot.txt plot; window size=3; score cut-off=3; from the peaks"
unknown_id EMBL/GenBank/SwissProt misc_binding 3034816 3034918 3.985935 + . note "Auto-generated from User algorithm from min_zscored_normalised_chIP_combined.plot.txt plot; window size=3; score cut-off=3; from the peaks"
unknown_id EMBL/GenBank/SwissProt misc_binding 3094306 3094355 4.129863 + . note "Auto-generated from User algorithm from min_zscored_normalised_chIP_combined.plot.txt plot; window size=3; score cut-off=3; from the peaks"
unknown_id EMBL/GenBank/SwissProt misc_binding 3234987 3235025 3.7350886 + . note "Auto-generated from User algorithm

from min_zscored_normalised_chIP_combined.plot.txt plot; window size=3; score cut-off=3; from the peaks"
unknown_id EMBL/GenBank/SwissProt misc_binding 3235116 3235187
3.69453 + . note "Auto-generated from User algorithm from min_zscored_normalised_chIP_combined.plot.txt plot; window size=3; score cut-off=3; from the peaks"
unknown_id EMBL/GenBank/SwissProt misc_binding 3287317 3287475
4.452784 + . note "Auto-generated from User algorithm from min_zscored_normalised_chIP_combined.plot.txt plot; window size=3; score cut-off=3; from the peaks"
unknown_id EMBL/GenBank/SwissProt misc_binding 3383662 3383700
3.8630922 + . note "Auto-generated from User algorithm from min_zscored_normalised_chIP_combined.plot.txt plot; window size=3; score cut-off=3; from the peaks"
unknown_id EMBL/GenBank/SwissProt misc_binding 3430382 3430418
5.1989303 + . note "Auto-generated from User algorithm from min_zscored_normalised_chIP_combined.plot.txt plot; window size=3; score cut-off=3; from the peaks"
unknown_id EMBL/GenBank/SwissProt misc_binding 3543799 3543842
4.1147494 + . note "Auto-generated from User algorithm from min_zscored_normalised_chIP_combined.plot.txt plot; window size=3; score cut-off=3; from the peaks"
unknown_id EMBL/GenBank/SwissProt misc_binding 3573834 3573897
4.5487356 + . note "Auto-generated from User algorithm from min_zscored_normalised_chIP_combined.plot.txt plot; window size=3; score cut-off=3; from the peaks"
unknown_id EMBL/GenBank/SwissProt misc_binding 3634252 3634288
3.9328818 + . note "Auto-generated from User algorithm from min_zscored_normalised_chIP_combined.plot.txt plot; window size=3; score cut-off=3; from the peaks"
unknown_id EMBL/GenBank/SwissProt misc_binding 3664731 3664769
3.6804066 + . note "Auto-generated from User algorithm from min_zscored_normalised_chIP_combined.plot.txt plot; window size=3; score cut-off=3; from the peaks"
unknown_id EMBL/GenBank/SwissProt misc_binding 3713810 3713851
4.2933784 + . note "Auto-generated from User algorithm from min_zscored_normalised_chIP_combined.plot.txt plot; window size=3; score cut-off=3; from the peaks"
unknown_id EMBL/GenBank/SwissProt misc_binding 3839587 3839718
8.007469 + . note "Auto-generated from User algorithm from min_zscored_normalised_chIP_combined.plot.txt plot; window size=3; score cut-off=3; from the peaks"
unknown_id EMBL/GenBank/SwissProt misc_binding 3908555 3908599
4.088568 + . note "Auto-generated from User algorithm from min_zscored_normalised_chIP_combined.plot.txt plot; window size=3; score cut-off=3; from the peaks"
unknown_id EMBL/GenBank/SwissProt misc_binding 3997560 3997610
5.4435315 + . note "Auto-generated from User algorithm

from min_zscored_normalised_chIP_combined.plot.txt plot; window size=3; score cut-off=3; from the peaks"
unknown_id EMBL/GenBank/SwissProt misc_binding 4011175 4011414 6.789148 + . note "Auto-generated from User algorithm from min_zscored_normalised_chIP_combined.plot.txt plot; window size=3; score cut-off=3; from the peaks"
unknown_id EMBL/GenBank/SwissProt misc_binding 4045027 4045147 6.6084704 + . note "Auto-generated from User algorithm from min_zscored_normalised_chIP_combined.plot.txt plot; window size=3; score cut-off=3; from the peaks"
unknown_id EMBL/GenBank/SwissProt misc_binding 4093598 4093634 3.8489091 + . note "Auto-generated from User algorithm from min_zscored_normalised_chIP_combined.plot.txt plot; window size=3; score cut-off=3; from the peaks"
unknown_id EMBL/GenBank/SwissProt misc_binding 4154724 4154883 5.947699 + . note "Auto-generated from User algorithm from min_zscored_normalised_chIP_combined.plot.txt plot; window size=3; score cut-off=3; from the peaks"
unknown_id EMBL/GenBank/SwissProt misc_binding 4158657 4158693 3.6208665 + . note "Auto-generated from User algorithm from min_zscored_normalised_chIP_combined.plot.txt plot; window size=3; score cut-off=3; from the peaks"
unknown_id EMBL/GenBank/SwissProt misc_binding 4417177 4417213 4.134798 + . note "Auto-generated from User algorithm from min_zscored_normalised_chIP_combined.plot.txt plot; window size=3; score cut-off=3; from the peaks"
unknown_id EMBL/GenBank/SwissProt misc_binding 4491925 4491962 4.8901234 + . note "Auto-generated from User algorithm from min_zscored_normalised_chIP_combined.plot.txt plot; window size=3; score cut-off=3; from the peaks"
unknown_id EMBL/GenBank/SwissProt misc_binding 4507569 4508036 16.233952 + . note "Auto-generated from User algorithm from min_zscored_normalised_chIP_combined.plot.txt plot; window size=3; score cut-off=3; from the peaks"
unknown_id EMBL/GenBank/SwissProt misc_binding 4511310 4511773 9.672637 + . note "Auto-generated from User algorithm from min_zscored_normalised_chIP_combined.plot.txt plot; window size=3; score cut-off=3; from the peaks"

Supplementary file 2

motif.gff

A general feature format (.gff) file indicating the position of motifs identified using YMF in gff. This file can be viewed in the Artemis or IGB genome browser software.

unknown_id	EMBL/GenBank/SwissProt	misc_binding	602085	602092	.	-	.	gene "motif_3.1" ; note "Peak height = 8.441328"
unknown_id	EMBL/GenBank/SwissProt	misc_binding	750349	750356	.	-	.	gene "motif_4.1" ; note "Peak height = 9.229065"
unknown_id	EMBL/GenBank/SwissProt	misc_binding	750356	750363	.	-	.	gene "motif_4.2" ; note "Peak height = 6.9578667"
unknown_id	EMBL/GenBank/SwissProt	misc_binding	985412	985419	.	-	.	gene "motif_1.1" ; note "Peak height = 13.692948"
unknown_id	EMBL/GenBank/SwissProt	misc_binding	985413	985420	.	+	.	gene "motif_1.2" ; note "Peak height = 13.860914"
unknown_id	EMBL/GenBank/SwissProt	misc_binding	1382277	1382284	.	+	.	gene "motif_1.3" ; note "Peak height = 11.438374"
unknown_id	EMBL/GenBank/SwissProt	misc_binding	1382437	1382444	.	-	.	gene "motif_3.2" ; note "Peak height = 3.5896993"
unknown_id	EMBL/GenBank/SwissProt	misc_binding	1840330	1840337	.	-	.	gene "motif_1.4" ; note "Peak height = 8.738397"
unknown_id	EMBL/GenBank/SwissProt	misc_binding	1840331	1840338	.	+	.	gene "motif_1.5" ; note "Peak height = 8.829524"
unknown_id	EMBL/GenBank/SwissProt	misc_binding	1840367	1840374	.	-	.	gene "motif_5.1" ; note "Peak height = 6.6060224"
unknown_id	EMBL/GenBank/SwissProt	misc_binding	2117842	2117849	.	-	.	gene "motif_1.6" ; note "Peak height = 7.733407"
unknown_id	EMBL/GenBank/SwissProt	misc_binding	2208684	2208691	.	-	.	gene "motif_1.7" ; note "Peak height = 5.138975"
unknown_id	EMBL/GenBank/SwissProt	misc_binding	2208685	2208692	.	+	.	gene "motif_1.8" ; note "Peak height = 5.2866807"
unknown_id	EMBL/GenBank/SwissProt	misc_binding	2208759	2208766	.	-	.	gene "motif_1.9" ; note "Peak height = 4.539592"
unknown_id	EMBL/GenBank/SwissProt	misc_binding	3094309	3094316	.	-	.	gene "motif_1.10" ; note "Peak height = 4.175324"
unknown_id	EMBL/GenBank/SwissProt	misc_binding	3094310	3094317	.	+	.	gene "motif_1.11" ; note "Peak height = 4.252162"
unknown_id	EMBL/GenBank/SwissProt	misc_binding	3235016	3235023	.	-	.	gene "motif_1.12" ; note "Peak height = 3.8112814"
unknown_id	EMBL/GenBank/SwissProt	misc_binding	4011263	4011270	.	-	.	gene "motif_4.3" ; note "Peak height = 7.111929"
unknown_id	EMBL/GenBank/SwissProt	misc_binding	4011295	4011302	.	+	.	gene "motif_3.3" ; note "Peak height = 9.476667"
unknown_id	EMBL/GenBank/SwissProt	misc_binding	4011316	4011323	.	-	.	gene "motif_1.13" ; note "Peak height = 9.581232"
unknown_id	EMBL/GenBank/SwissProt	misc_binding	4011317	4011324	.	+	.	gene "motif_1.14" ; note "Peak height = 9.437758"
unknown_id	EMBL/GenBank/SwissProt	misc_binding	4045077	4045084	.	+	.	gene "motif_1.15" ; note "Peak height = 8.977181"
unknown_id	EMBL/GenBank/SwissProt	misc_binding	4154806	4154813	.	-	.	gene "motif_1.16" ; note "Peak height = 6.9492903"
unknown_id	EMBL/GenBank/SwissProt	misc_binding	4154807	4154814	.	+	.	gene "motif_1.17" ; note "Peak height = 6.930189"

unknown_id EMBL/GenBank/SwissProt misc_binding 4507573 4507580
. + . gene "motif_4.4" ; note "Peak height = 6.9181685"
unknown_id EMBL/GenBank/SwissProt misc_binding 4507613 4507620
. + . gene "motif_3.4" ; note "Peak height = 18.59982"
unknown_id EMBL/GenBank/SwissProt misc_binding 4507698 4507705
. - . gene "motif_2.1" ; note "Peak height = 12.936734"
unknown_id EMBL/GenBank/SwissProt misc_binding 4507829 4507836
. + . gene "motif_1.18" ; note "Peak height = 42.443794"
unknown_id EMBL/GenBank/SwissProt misc_binding 4507951 4507958
. - . gene "motif_4.5" ; note "Peak height = 8.355333"
unknown_id EMBL/GenBank/SwissProt misc_binding 4507995 4508002
. - . gene "motif_5.2" ; note "Peak height = 5.989598"
unknown_id EMBL/GenBank/SwissProt misc_binding 4511433 4511440
. - . gene "motif_1.19" ; note "Peak height = 14.4042015"
unknown_id EMBL/GenBank/SwissProt misc_binding 4511434 4511441
. + . gene "motif_1.20" ; note "Peak height = 14.597094"
unknown_id EMBL/GenBank/SwissProt misc_binding 4511480 4511487
. + . gene "motif_1.21" ; note "Peak height = 18.152327"
